# Supplementary material for: Genome-Wide Analysis of the SNARE Family in Cultivated Peanut (Arachis hypogaea L.) Reveals That Some Members Are Involved in Stress Responses
Source: Int J Mol Sci. 2023 Apr 12;24(8):7103. doi: 10.3390/ijms24087103 (PMC10139436; doi:10.3390/ijms24087103)
Supplement: Supplementary file 1 [file ijms-24-07103-s001.zip › Table S1.docx]

| Annotion | Sense primer(5’-3’) | Antisense primer(5’-3’) |
| --- | --- | --- |
| SYP122a | TTGACCTTCTCATCTCTA | CTGAATCTCTTGAATTGTG |
| SYP132a | GAGCGAGGATGAATATGA | TGACTGTAATAACTCTTCTCT |
| SYP22a | AAGAGATCAAGGCATTCAAG | ACCCAATATCATCAATCATAGC |
| NPSN12b | CTCTATTCAGTTCTCAATTAAG | GATTACACCACAGACAAT |
| GOS12a | GCTACTTCTGTAACTCAA | TGTCTTAATATCAGTAATATCATC |
| SNAP33a | TTCATCTTACGCCTATTC | AACAGCATAATTCTCCAA |
| SFT12a | AGATAGGCACAGAAGTAA | AATGTAACGATATGAAGAATG |
| BET12b | TCAATCTCTCTACTCTTCTT | CACCTCCTCCTGTATATC |
| VAMP725 | ACTGAGAACCTTCACTAT | ATTATGATGGCAAGAACTAT |
| VAMP721a | CAACAACAAGTTCACCTACA | TCTTCCGACCGATTCATC |
| SYP122a Full | AATAACACAACCACCCCAACAA | TCAATAACACAACCACCCCAAC |
| SNAP33a Full | TTGATTACCTGATGAGTGTGC | CAGGGTAGGAAGGAAACAATA |
| VAMP721a Full | AACAAAACACAAGTCACAGCC | CTTGTCACATTATTTCTCCCTT |

Table S1 Primer pairs for qPCR and full length coding sequence
